# The Role of Gasdermin B-Mediated Pyroptosis in Bladder Cancer Diagnosis

**DOI:** 10.3390/ijms27083540

**Published:** 2026-04-16

**Authors:** Sara Pączek, Michał Olkowicz, Jacek Kudelski, Monika Gudowska-Sawczuk

**Affiliations:** 1 Department of Biochemical Diagnostics, University Hospital of Bialystok, Waszyngtona 15A St., 15-269 Bialystok, Poland; sara.paczek@uskwb.pl; 2 Department of Urology, University Hospital of Bialystok, M. Skłodowskiej-Curie 24A St., 15-276 Bialystok, Poland; m.olkowicz@yahoo.com; 3 Department of Urology, Medical University of Bialystok, M. Skłodowskiej-Curie 24A St., 15-276 Bialystok, Poland; jkudelski@op.pl; 4 Department of Biochemical Diagnostics, Medical University of Bialystok, Waszyngtona 15A St., 15-269 Bialystok, Poland

**Keywords:** gasdermin B, GSDM B, pyroptosis, bladder cancer, diagnosis, marker

## Abstract

Bladder cancer (BC) is one of the most common urinary tract malignancies. In recent years, increasing attention has been paid to the role of pyroptosis—an inflammatory form of programmed cell death—in cancer development. Gasdermin B (GSDM B), a member of the gasdermin protein family, is involved in the regulation of inflammatory processes and the immune response, and its expression may be associated with cancer development and progression. The aim of the study was to assess GSDM B concentrations in the serum of patients with bladder cancer and to determine its potential diagnostic value in comparison with the tumor markers carcinoembryonic antigen (CEA) and carbohydrate antigen 19-9 (CA19-9). This study included patients with bladder cancer hospitalized at the Department of Urology, Medical University of Białystok, and a healthy control group. GSDM B concentrations were determined by Enzyme-Linked Immunosorbent Assay (ELISA), while CEA and CA19-9 concentrations were determined by chemiluminescent microparticle immunoassay (CMIA). Concentrations in the serum of patients with bladder cancer were significantly higher than in the control group. A positive correlation was found between GSDM B and CEA and CA19-9 concentrations, as well as the age of the subjects. Receiver-operating characteristic (ROC) analysis demonstrated moderate but significant diagnostic value of GSDM B in differentiating patients with BC from healthy controls. No significant differences in GSDM B concentrations were observed between low- and high-grade tumors. These findings suggest that GSDM B may serve as a potential diagnostic marker for bladder cancer, particularly when used as part of a multimarker panel.

## 1. Introduction

Urological cancers constitute a significant global health burden, with bladder cancer (BC) and renal cell carcinoma (RCC) being among the most prevalent malignancies in this category. These cancers originate in different sections of the urinary tract and exhibit diverse histological characteristics and molecular mechanisms [1,2].

BC ranks as the 10th most common cancer worldwide, with approximately 550,000 new cases diagnosed annually [3]. Histologically, the predominant subtype is urothelial carcinoma, accounting for over 90% of cases. Squamous cell carcinoma (SCC), adenocarcinoma (AC), and small cell carcinoma are less common. In terms of the gender distribution, BC occurs approximately four times more frequently in men than women [4].

Urological cancers are influenced by multiple risk factors, including lifestyle choices, environmental exposures, genetic predispositions, and infections. Tobacco smoking remains the leading cause of many cancers including BC, responsible for 50–65% of cases in men and 20–30% in women, as carcinogenic toxins accumulate in the urine and damage the bladder epithelium. Additional risk factors include occupational carcinogen exposure, chronic urinary tract infections, and genetic predispositions [5,6].

Symptoms of urological cancers often appear at advanced stages, with hematuria being the most common clinical presentation. Other symptoms include dysuria, flank pain, urinary urgency, weight loss, and fatigue, which vary depending on tumor location and stage [7]. Cystoscopy with biopsy remains the gold standard for diagnosing bladder cancer, while ultrasound, computed tomography (CT), and magnetic resonance imaging (MRI) are frequently used for RCC detection. Innovations in diagnostics, such as liquid biopsies and urinary biomarkers, are being explored to improve early detection and risk stratification. Treatment modalities for urological cancers are determined by disease stage and histology [8,9].

Gasdermin B (GSDM B) is a member of the gasdermin family, which is involved in pyroptosis—the inflammatory form of programmed cell death. This family of pore-forming proteins also plays a crucial role in regulation of immune responses and inflammation. The gasdermin group comprises six members: GSDM A, GSDM B, GSDM C, GSDM D, GSDM E and GSDM F [10]. These molecules are best known for mediating pyroptosis, a highly inflammatory form of programmed cell death. This process is initiated when immune cells detect the cellular damage of pathogen-associated signals, triggering the activation of inflammasomes [11]. As a result, membrane pores are formed, facilitating the release of pro-inflammatory cytokines such as IL-1β, which in consequence recruit and activate other immune cells at the site of infection. However, beyond their role in infection, gasdermins are also implicated in various conditions such as gastrointestinal diseases and cancer [12]. Their role in cancer depends on the context, as they have the ability to show both tumor-promoting and tumor-suppressing effects [13]. The tumor-promoting features have been investigated in breast cancer, where the overexpression of GSDM B was associated with human epidermal growth factor receptor 2 (HER-2)-positive breast cancers. It also correlated with poor prognosis as well as the presence of metastases [14]. In a certain context, GSDM B can be cleaved by Granzyme A (GZM A) to trigger the pyroptosis in cancer cells. Therefore, cytotoxic immune cells that infiltrate tumors and express GZM A can activate GSDM B, leading to inflammatory cell death in cancer cells [15]. GSDM B plays a multifaceted role in urological cancers, including bladder cancer. In conclusion, GSDM B seems to be a pivotal regulator in bladder cancer with the influence on tumor metabolism, immune response and the treatment outcomes. Its dual role in promoting tumor progression and mediating immune cell-induced death makes it a promising target for therapeutic interventions. Furthermore, understanding the regulatory mechanisms governing gasdermin activation and their various interactions with other cellular pathways will be crucial for the development of targeted therapies that can either harness or inhibit their activity as needed. However, little is currently known about their potential role as bladder cancer biomarkers. Therefore, the aim of this study was to evaluate the significance of GSDM B concentrations in the serum of patients with bladder cancer.

## 2. Results

**Figure 1:**
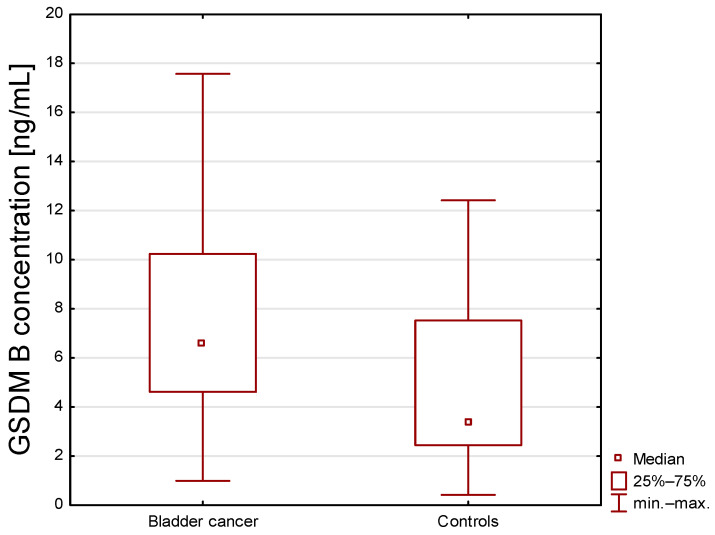
Serum concentration of gasdermin B in bladder cancer patients and healthy subjects. Statistical differences between groups are assessed using the U Mann–Whitney test. The difference in GSDM B concentrations between the Control and Bladder cancer patients is statistically significant (*p* < 0.001).

The mean serum concentration of GSDM B in patients with BC was significantly higher than in control subjects (mean (min–max values): 9.16 (0.99–40.49) ng/mL vs. 4.69 (0.42–12.42) ng/mL) (*p*  <  0.001) (Figure 1).

Among the other analyzed parameters, C-reactive protein and well-characterized tumor markers (CEA and CA19-9) differed significantly between patients with bladder cancer and healthy volunteers (*p* < 0.001; *p* < 0.001; *p* = 0.037, respectively). The serum concentration of both creatinine and urea was similar between the tested groups (*p* = 0.350 and *p* = 0.081, respectively). The results are presented in Table 1.

**Figure 2:**
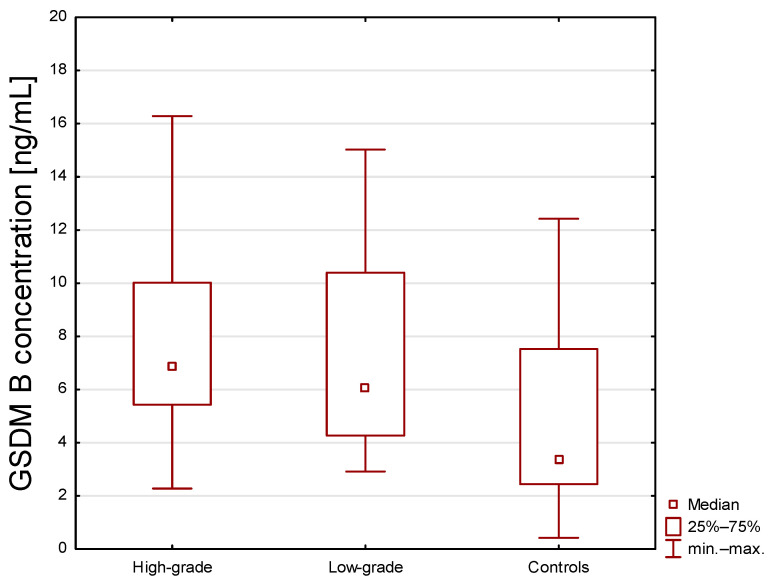
Serum concentrations of gasdermin B in high-grade, low-grade bladder cancer patients and healthy subjects. Statistical differences between groups were assessed using the Kruskal–Wallis ANOVA test. Statistically significant differences are observed between low-grade vs. controls (*p* = 0.014) and high-grade vs. controls (*p* = 0.003), but not between high-grade vs. low-grade (*p* = 1.000).

We also evaluated the concentrations of tested variables according to tumor grade. We found that the concentrations of GSDM B were different between the tested groups (*p* = 0.001, H = 13.330). The concentration of GSDM B in healthy subjects was significantly lower in comparison to low-grade (mean (min–max values): 8.15 (2.92–30.50) ng/mL; *p* = 0.014) and high-grade cancer (mean (min–max values): 10.38 (2.28–40.49) ng/mL; *p* = 0.003). The difference between low- and high-grade was not statistically significant (*p* = 1.000) (Figure 2).

The concentrations of CRP, CEA, urea and creatinine differ significantly between tested subgroups (*p* < 0.001, H = 18.325; *p* < 0.001, H = 21.213; *p* = 0.007, H = 9.943; *p* = 0.037, H = 6.588).

The concentrations of CRP and CEA in the control group were significantly lower than in low- (*p* = 0.012; *p* < 0.001, respectively) and high-grade BC (*p* < 0.001; *p* = 0.001). The concentration of urea was statistically higher in high-grade BC in comparison to low-grade (*p* = 0.020) and controls (0.013). The concentration of creatinine was lower in low-grade compared to high-grade cancer (*p* = 0.050). The concentrations of CA19-9 did not differ between groups (*p* = 0.136, H = 3.994) (Table 2).

Kruskal–Wallis ANOVA analysis showed that there were no statistically significant differences in GSDM B (*p* = 0.311, H = 2.335), CEA (*p* = 0.292, H = 2.464) or CA19-9 (*p* = 0.092, H = 4.777) concentrations according to tumor invasion depth (Table 3).

GSDM B concentrations were compared between females (mean ± SD: 6.086 ± 6.198 ng/mL) and males (mean ± SD: 8.129 ± 7.469 ng/mL) in the entire study population, and no statistically significant differences were observed (*p* = 0.054).

Correlations of GSDM B with other tested parameters are presented in Table 4. Spearman’s rank correlation test demonstrated the strongest positive correlation between the concentration of GSDM B and the CEA (r = 0.764). Moreover, GSDM B correlated with CA19-9. Positive correlations were observed between GSDM B and age, and between CRP and age. CRP concentration showed a significant positive correlation with CEA concentration, while CEA also correlated with CA19-9 and age. Creatinine concentration correlated with urea only. No significant negative correlations were observed.

Multivariate analysis was performed using binary logistic regression (generalized linear model, binomial distribution with logit link function). GSDM B, age, and CEA were all independent predictors of bladder cancer (*p* = 0.04, *p* < 0.05, and *p* = 0.02, respectively).

**Figure 3:**
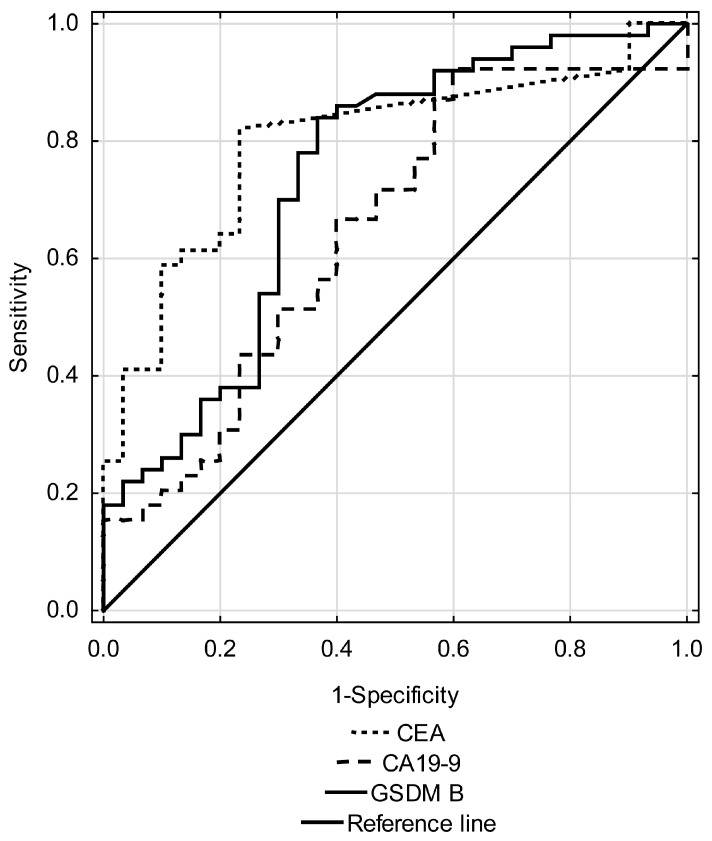
ROC curves for GSDM B, CEA, and CA19-9 in bladder cancer.

The results of the diagnostic significance of GSDM B, CEA and CA19-9 are summarized in Table 5. Analysis of ROC curve (Figure 3) revealed that GSDM B has moderate, but significant diagnostic value. The optimal cut-off point was set at 4.17 ng/mL, at which a sensitivity of 84.0% and a specificity of 63.3% were obtained, which indicates a high value in distinguishing bladder cancer patients from healthy individuals. The PPV was 77.6% and the negative NPV was 70.4%, resulting in an overall ACC of 76.3%. Compared with already known tumor markers such as CEA and CA19-9, GSDM B achieves diagnostic performance similar to CEA and outperforms CA19-9 in terms of overall accuracy.

## 3. Discussion

Pyroptosis, a form of programmed cell death, is an actively induced cell death, which involves the breakdown of the cell membrane and its disruption, leading to the release of intracellular molecules, including proinflammatory cytokines (IL-1β, IL-18). Pyroptosis plays an important role in the body’s immune response against intracellular pathogens; however, its excessive activation may contribute to the exacerbation of inflammation and tissue damage [16,17,18]. One of the gasdermin family proteins that can form pores in the cell membrane upon activation, leading to pyroptosis, is GSDM B. In brief, like other gasdermins, GSDM B consists of an N-terminal effector domain and a C-terminal domain that inhibits its activity. Upon cleavage of the protein by cytotoxic proteases, primarily granzyme A, the N-domain is released and inserts into the cell membrane, forming pores and leading to cell lysis and pyroptosis [19]. It has been demonstrated that the action of GSDM B may exert a dual effect on cancer development. On the one hand, pyroptosis causes the elimination of cancer cells, while on the other, overexpression of GSDM B may increase the invasiveness and metastasis of cancer cells [20]. However, the role of GSDM B is not exclusively tumor-suppressive. Under conditions of chronic activation, pyroptosis may instead promote a pro-tumorigenic microenvironment. Persistent inflammatory signaling, driven by cytokines such as interleukin-1β and interleukin-18, can support tumor proliferation, angiogenesis, and immune evasion. Therefore, GSDM B functions as a molecular switch that can either facilitate tumor cell elimination or contribute to cancer progression, depending on the balance between effective immune activation and chronic inflammation [21,22].

To date, only a few studies have investigated the significance of GSDM B in the development of bladder cancer. Overexpression of GSDM B mRNA level in bladder cancer cells was observed. It has been suggested that increased expression of GSDM B is responsible for facilitating the tumor cancer growth via activation of STAT3 signaling. On the other hand, it has been shown that USP24-mediated stabilization of GSDM B activates STAT3 signaling, an effect that can be blocked by the USP24 inhibitor [23]. Therefore, it seems that GSDM B may be associated with the development of bladder cancer—both as a factor promoting progression and as a potential diagnostic and prognostic biomarker.

Based on the above findings, the aim of our study was to evaluate the diagnostic utility of GSDM B in bladder cancer. Our study showed that, in patients with bladder cancer, the serum concentration of GSDM B was significantly higher—nearly twice as high—as in healthy individuals (9.16 ng/mL vs. 4.69 ng/mL). Elevated expression of GSDM B in cancer cells may lead to increased release of the protein into the bloodstream, which could explain the elevated serum concentrations of GSDM B observed in patients with BC. Furthermore, the demonstrated positive correlation with CRP may further support the association of this protein with inflammation and carcinogenesis. Interestingly, the positive correlations with tumor markers CEA and CA19-9 suggest that GSDM B may likely reflect similar biological mechanisms, such as tumor cell proliferation. In contrast, the lack of correlation with urea and creatinine indicates that GSDM B is of neoplastic, not metabolic, origin. These findings suggest that renal clearance does not significantly influence GSDM B concentrations. Moreover, we evaluated GSDM B as a potential diagnostic biomarker for bladder cancer using multivariate binary logistic regression analysis. We demonstrated that GSDM B, age as well as CEA were independent predictors of BC. The independent association of GSDM B with bladder cancer suggests that it may play a role in tumor-related biological processes rather than being solely dependent on age or overall tumor burden, as reflected by CEA levels.

On the other hand, statistical analysis according to stage of advancement showed that GSDM B concentration was lower in healthy individuals compared to low- and high-grade BC, but without significant difference between individual subgroups of BC patients. The lack of difference between LG and HG tumors may be due to the fact that GSDM B overexpression occurs early in the development of cancer and further increases in malignancy do not lead to a significant increase in its level. Furthermore, it appears that GSDM B may indeed play a dual role in the initiation and progression of tumors: pro- and antiangiogenic. Therefore, it is difficult to determine whether the increase in GSDM B is the cause or the consequence of the presence of bladder cancer.

ROC curve analysis showed that GSDM B concentration had moderate but significant diagnostic value. The area under the curve (AUC) for GSDM B was 0.736 ± 0.061, indicating good ability to discriminate between diseased and healthy individuals. The optimal cut-off point was set at 4.17 ng/mL. Compared with classical markers such as CEA and CA19-9, GSDM B achieves diagnostic performance similar to CEA and outperforms CA19-9 in overall accuracy. Therefore, it may represent a promising complementary biomarker, especially in multimarker strategies that increase diagnostic sensitivity. Considering the choice of serum over urine as the primary sample, even though urine has direct contact with the bladder epithelium, we chose serum because of its stability and the ability to compare results with other blood-based tumor markers, such as CEA and CA19-9, which is worth emphasizing. GSDM B in urine remains a promising avenue for future research, as direct tumor-to-urine contact may potentially increase diagnostic yield. The aim of our study was to collect baseline data on GSDM B in serum, providing a starting point for future urinary biomarker research.

Given the limitations, the relatively small sample size of the study should be noted, which may affect the generalizability of the results and the precision of diagnostic estimates, such as specificity and sensitivity. The diagnostic performance of serum GSDM B, with an AUC of 0.736, is moderate, suggesting limited discriminatory power when used as a standalone marker. However, GSDM B may therefore be a useful complementary diagnostic tool, particularly in combination with established markers such as CEA. The lack of differences between low- and high-grade tumors limits its prognostic value, suggesting that GSDM B may be more suitable for diagnostic purposes than for predicting disease progression or prognosis. A limitation of this study is the lack of external validation in an independent patient cohort. Therefore, further studies with larger, multicenter cohorts and urine samples are needed to better elucidate the diagnostic and clinical utility of GSDM B in bladder cancer.

## 4. Materials and Methods

### 4.1. Study Design

This is an experimental study that evaluates the concentration of gasdermin B as well as comparative biomarkers CEA and CA19-9 in patients with bladder cancer.

### 4.2. Bioethics Committee

This study was conducted at the Medical University of Bialystok and received approval from the Medical University of Bialystok Bioethical Committee (approval number APK.002.240.220).

### 4.3. Blood Samples

All samples were obtained by venipuncture from the healthy participants and bladder cancer patients before TURBT procedure. The collected blood samples were centrifuged to separate the serum. Serum samples were stored at −80 °C until analysis.

### 4.4. Participants

**Figure 4:**
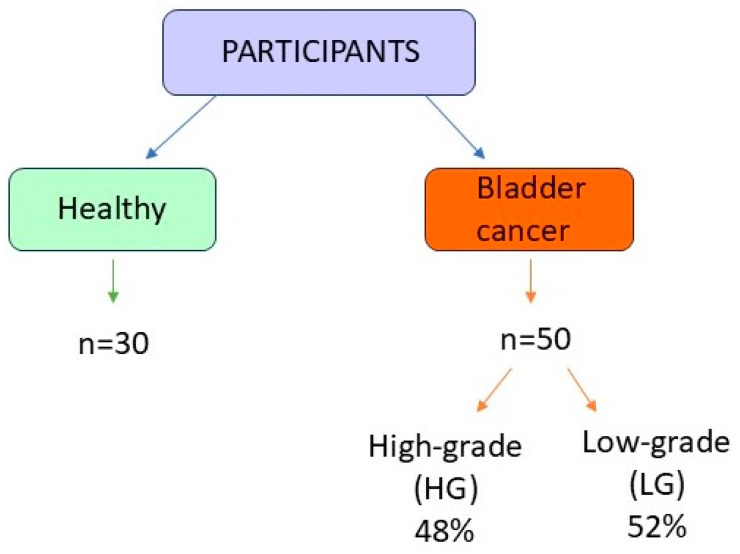
The summary of tested groups.

The summary of tested groups is presented in Figure 4. Aside from the classification into low- and high-grade tumors, patients were also classified based on the depth of tumor invasion (T) into three groups: Ta (50%), T1 (40.9%), and T2 (9.1%). The BC group comprised 8 females and 42 males (42–87 years). All BC patients were admitted to the Urology Department of the Medical University of Bialystok, University Hospital of Bialystok. The diagnosis was made on the basis of ultrasound, cystoscopy, symptoms, and the results of histopathological examination. All patients were scheduled for TURBT—transurethral resection of bladder tumor. No other active cancer or acute inflammation was detected in any of the patients.

The control group was aged 17–79 and comprised 17 females and 13 males. Exclusions for inclusion in the control group included: pathological conditions of the urinary system and acute inflammation.

### 4.5. Methods

The concentrations of all tested parameters were determined in serum and in accordance with the manufacturer’s guidelines. GSDM B determinations were made in samples diluted twofold. The details of the applied methods are presented in Table 6.

### 4.6. Statistical Analysis

Statistical analyses were performed with Statistica 13.3 software. Statistical significance was determined using the Mann–Whitney U and ANOVA tests to evaluate differences between tested groups. The Spearman rank correlation test was used to check the relationship between tested variables. Multivariate analysis was performed using binary logistic regression (generalized linear model, binomial distribution with logit link function) to identify independent predictors of BC. Results are shown as the mean ± SD. Differences were considered significant at *p* < 0.05.

## 5. Conclusions

The results of our analysis suggest that GSDM B may serve as a potential diagnostic marker for bladder cancer, especially when used in combination with CEA. High sensitivity with moderate specificity suggests that elevated serum GSDM B levels may reflect the presence of cancer. However, the lack of differences between low- and high-grade GSDM B limits its prognostic value. GSDM B may therefore be a useful complementary diagnostic tool, but it does not appear to be a predictor of disease outcome.

## Figures and Tables

**Table 1:** The results of CEA, CA19-9, CRP, creatinine and urea in the tested groups. Tested parameters: Mean (min–max values).

	CEA [ng/mL]	CA19-9 U/mL	CRP mg/dL	Creatinine mg/dL	Urea mg/dL
Controls (A)	1.87 *^B^ (0.59–4.26)	6.12 *^B^ (2.06–28.40)	1.97 *^B^ (1.00–6.70)	0.81 (0.55–1.04)	30.84 (12.84–49.92)
Bladder cancer (B)	4.00 *^A^ (1.72–18.83)	18.03 *^A^ (2.05–256.43)	24.85 *^A^ (1.00–103.00)	0.89 (0.51–2.13)	37.36 (17.12–107.00)

^A^, controls; ^B^, bladder cancer. *—the significant differences between groups.

**Table 2:** The results of CEA, CA19-9, CRP, creatinine and urea according to tumor grade. Tested parameters: Mean (min–max values).

	CEA [ng/mL]	CA19-9 U/mL	CRP mg/dL	Creatinine mg/dL	Urea mg/dL
Low-grade (L)	3.75 (1.73–12.16)	9.57 (2.05–74.06)	29.52 (1.00–103.00)	0.81 (0.51–1.16)	30.85 (17.12–47.08)
High-grade (H)	4.61 (1.73–18.83)	29.94 (17.12–79.18)	18.35 (1.10–103.00)	0.99 (0.61–2.13)	41.84 (17.12–79.18)

L, low-grade; H, high-grade.

**Table 3:** GSDM B, CEA and CA19-9 concentrations according to tumor invasion depth (T stage). Tested parameters: Mean (min-max values).

	GSDM B [ng/mL]	CEA [ng/mL]	CA19-9 U/mL
Ta	8.09 (2.28–30.50)	3.77 (1.72–12.16)	9.30 (2.05–74.06)
T1	11.63 (2.69–40.49)	6.13 (1.73–18.83)	43.73 (2.05–256.43)
T2	6.43 (4.70–8.27)	2.36 (1.73–3.00)	9.51 (2.12–29.83)

**Table 4:** Spearman’s correlations between tested variables in the total study group.

	Correlation Ratio (r)						
	0.000–0.100						
	0.101–0.300						
	0.301–0.500						
	0.501–0.700						
	0.701–0.900						
Total study group	GSDM B	CRP	CEA	CA19-9	Creatinine	Urea	Age
GSDM B							
r		0.179	0.764	0.263	−0.055	0.165	0.426
*p*		0.129	<0.001 *	0.029 *	0.626	0.154	<0.001 *
CRP							
r	0.179		0.352	0.099	0.053	0.146	0.407
*p*	0.129		0.005 *	0.443	0.656	0.219	<0.001 *
CEA							
r	0.764	0.352		0.269	−0.053	0.168	0.367
*p*	<0.001 *	0.005 *		0.026 *	0.660	0.177	0.002 *
CA19-9							
r	0.263	0.099	0.269		0.118	0.289	0.291
*p*	0.029 *	0.443	0.026 *		0.336	0.019 *	0.015 *
Creatinine							
r	−0.055	0.053	−0.053	0.118		0.596	0.129
*p*	0.626	0.656	0.660	0.336		<0.001 *	0.251
Urea							
r	0.165	0.146	0.168	0.289	0.596		0.404
*p*	0.154	0.219	0.177	0.019 *	<0.001 *		<0.001 *
Age							
r	0.426	0.407	0.367	0.291	0.129	0.404	
*p*	<0.001 *	<0.001 *	0.002 *	0.015 *	0.251	<0.001 *	

* indicates a statistically significant correlation.

**Table 5:** The diagnostic significance of serum GSDM B, CEA and CA19-9 in bladder cancer.

	Cut-Off from the ROC	AUC	SE	Sensitivity [%]	Specificity [%]	PPV [%]	NPV [%]	ACC [%]
GSDM B [ng/mL]	4.17	0.736	0.061	84.0	63.3	77.6	70.4	76.3
CEA [ng/mL]	1.74	0.770	0.055	82.1	76.7	82.1	76.7	79.7
CA19-9 [U/mL]	2.07	0.648	0.068	92.3	40.0	66.7	80.0	69.6

AUC, area under the curve; SE, standard error; PPV, positive predictive value; NPV, negative predictive value; and ACC, accuracy.

**Table 6:** Details of applied methods.

Category	Parameter	Analyzer	Unit	Detection Range	Method
immunochemistry	GSDM B	ETI-max 3000 (DiaSorin Inc., Stillwater, USA)	ng/mL	0.32–20.00	ELISA
immunochemistry	CEA	Alinity ci (Abbott, Illinois, USA)	ng/mL	1.73–1500.00	CMIA
immunochemistry	CA 19-9	Alinity ci (Abbott, Illinois, USA)	U/mL	2.06–1200.00	CMIA
biochemistry	CRP	Alinity c (Abbott, Illinois, USA)	mg/dL	1.00–480.00	immunoturbidimetric
biochemistry	Urea	Alinity c (Abbott, Illinois, USA)	mg/dL	3.00–125.00	enzymatic
biochemistry	Creatinine	Alinity c (Abbott, Illinois, USA)	mg/dL	2.5–400.00	enzymatic

## Data Availability

The data that support the findings will be available upon request under the corresponding author’s e-mail: monika.gudowska-sawczuk@umb.edu.pl.

## References

[1] Mourão T.C., Curado M.P., de Oliveira R.A.R., Santana T.B.M., Favaretto R.L., Guimarães G.C. (2022). Epidemiology of Urological Cancers in Brazil: Trends in Mortality Rates Over More Than Two Decades. J. Epidemiol. Glob. Health.

[2] Barber N., Ali A. (2022). Urologic Cancers.

[3] Richters A., Aben K.K.H., Kiemeney L.A.L.M. (2020). The global burden of urinary bladder cancer: An update. World J. Urol..

[4] Downes M.R., Barber N., Ali A. (2022). Invasive Urothelial Carcinoma: Subtypes and Divergent Differentiation. Urologic Cancers.

[5] Freedman N.D., Silverman D.T., Hollenbeck A.R., Schatzkin A., Abnet C.C. (2011). Association between smoking and risk of bladder cancer among men and women. JAMA.

[6] Alouini S. (2024). Risk Factors Associated with Urothelial Bladder Cancer. Int. J. Environ. Res. Public Health.

[7] Leslie S.W., Soon-Sutton T.L., Aeddula N.R. (2025). Bladder Cancer. StatPearls.

[8] Abouelkheir R.T., Abdelhamid A., Abou El-Ghar M., El-Diasty T. (2021). Imaging of Bladder Cancer: Standard Applications and Future Trends. Medicina.

[9] Zhu C.Z., Ting H.N., Ng K.H., Ong T.A. (2019). A review on the accuracy of bladder cancer detection methods. J. Cancer.

[10] Slaufova M., Karakaya T., Di Filippo M., Hennig P., Beer H.D. (2023). The gasdermins: A pore-forming protein family expressed in the epidermis. Front. Immunol..

[11] Broz P. (2025). Pyroptosis: Molecular mechanisms and roles in disease. Cell Res..

[12] Liu X., Xia S., Zhang Z., Wu H., Lieberman J. (2021). Channelling inflammation: Gasdermins in physiology and disease. Nat. Rev. Drug Discov..

[13] Wang M., Chen X., Zhang Y. (2021). Biological Functions of Gasdermins in Cancer: From Molecular Mechanisms to Therapeutic Potential. Front. Cell Dev. Biol..

[14] Gámez-Chiachio M., Molina-Crespo Á., Ramos-Nebot C., Martinez-Val J., Martinez L., Gassner K., Llobet F.J., Soriano M., Hernandez A., Cordani M. (2022). Gasdermin B over-expression modulates HER2-targeted therapy resistance by inducing protective autophagy through Rab7 activation. J. Exp. Clin. Cancer Res..

[15] Lin W., Lin B., Zhou Q., Teng L. (2024). Gasdermin-mediated pyroptosis confers anticancer immunity. J. Immunother. Cancer.

[16] Wei Y., Yang L., Pandeya A., Cui J., Zhang Y., Li Z. (2022). Pyroptosis-Induced Inflammation and Tissue Damage. J. Mol. Biol..

[17] Zhang J., Wirtz S. (2022). Does Pyroptosis Play a Role in Inflammasome-Related Disorders?. Int. J. Mol. Sci..

[18] Sun J., Li Y. (2022). Pyroptosis and respiratory diseases: A review of current knowledge. Front. Immunol..

[19] Chen Q., Shi P., Wang Y., Zou D., Wu X., Wang D., Hu Q., Zou Y., Huang Z., Ren J. (2019). GSDM B promotes non-canonical pyroptosis by enhancing caspase-4 activity. J. Mol. Cell Biol..

[20] Li L., Li Y., Bai Y. (2020). Role of GSDM B in Pyroptosis and Cancer. Cancer Manag. Res..

[21] Fang Y., Tian S., Pan Y., Li W., Wang Q., Tang Y., Yu T., Wu X., Shi Y., Ma P. (2020). Pyroptosis: A new frontier in cancer. Biomed. Pharmacother..

[22] Xia X., Wang X., Cheng Z., Qin W., Lei L., Jiang J., Hu J. (2019). The role of pyroptosis in cancer: Pro-cancer or pro-”host”?. Cell Death Dis..

[23] He H., Yi L., Zhang B., Yan B., Xiao M., Ren J., Zi D., Zhu L., Zhong Z., Zhao X. (2021). USP24-GSDM B complex promotes bladder cancer proliferation via activation of the STAT3 pathway. Int. J. Biol. Sci..

